# Soft X-ray absorption spectroscopy in the low-energy region explored using an argon gas window

**DOI:** 10.1107/S1600577520005883

**Published:** 2020-05-27

**Authors:** Masanari Nagasaka

**Affiliations:** a Institute for Molecular Science, Myodaiji, Okazaki 444-8585, Japan; b SOKENDAI (The Graduate University for Advanced Studies), Myodaiji, Okazaki 444-8585, Japan

**Keywords:** soft X-ray absorption spectroscopy, low-energy region, argon gas window, atmospheric conditions

## Abstract

The soft X-ray region below 200 eV is important for investigating chemical and biological phenomena since it covers *K*-edges of Li and B and *L*-edges of Si, P, S and Cl. Helium gas is generally used as the soft X-ray transmission window for soft X-ray absorption spectroscopy (XAS) under atmospheric conditions. However, the helium gas window cannot be applied to XAS in the low-energy region since transmitted soft X-rays mostly consist of high-order X-rays due to the low transmission of first-order X-rays. In this study, the argon gas window is proposed as a new soft X-ray transmission window in the low-energy region. High-order X-rays are removed by the absorption of the Ar *L*-edge (240 eV), and first-order X-rays become the major contribution of transmitted soft X-rays in the low-energy region. Under atmospheric argon conditions, the double-excitation Rydberg series of helium gas (60 eV), Si *L*-edge XAS of an Si_3_N_4_ membrane (100 eV) and S *L*-edge XAS of di­methyl sulfoxide gas (170 eV) are successfully measured, indicating that the argon gas window is effective for soft X-ray transmission in the low-energy region from 60 eV to 240 eV.

## Abstract 2

Argon gas windows are shown to be effective for soft X-ray absorption spectroscopy in the low-energy region below 200 eV under atmospheric conditions.

## 1. Introduction   

Soft X-ray absorption spectroscopy (XAS) is an element-specific method used to investigate local electronic structures of materials since it observes the excitation process of a core electron to unoccupied orbitals (Stöhr, 1992 ▸). Since soft X-rays are strongly absorbed by air and liquids, XAS spectra of gas and solid samples under ultrahigh-vacuum conditions have been studied extensively. Recently, several groups have measured XAS spectra under atmospheric conditions using the high soft X-ray transmission of helium gas (Chantler, 2000 ▸). A differential pumping system is used between a soft X-ray beamline under ultrahigh-vacuum conditions and a measurement chamber under atmospheric helium conditions (Lee *et al.*, 2001 ▸; Gog *et al.*, 2007 ▸; Tamenori, 2010 ▸). X-ray photoelectron spectroscopy has also been developed for the measurement of samples near ambient atmospheric pressure (Takagi *et al.*, 2017 ▸), and is applied to the analyses of catalytic reactions (Kondoh *et al.*, 2016 ▸) and fuel cells (Takagi *et al.*, 2018 ▸).

As discussed above, the helium gas window is generally used for soft X-ray transmission. The water window at the energy region from the C *K*-edge (280 eV) to the O *K*-edge (530 eV) is widely used for microscopic measurements of biomaterials in aqueous solutions (Ford *et al.*, 1991 ▸) and ultrafast spectroscopy using high harmonic generation (Spielmann, 1997 ▸; Takahashi *et al.*, 2008 ▸). Recently, XAS has been applied extensively to liquids and solutions in the soft X-ray region (Smith & Saykally, 2017 ▸). Si_3_N_4_ membranes with a thickness of 100 nm are widely used for XAS of liquid samples in transmission mode (Yang & Kirz, 1987 ▸). Several groups measured XAS of liquid samples in transmission mode by packing liquid samples with Si_3_N_4_ membranes (Näslund *et al.*, 2005 ▸; Schreck *et al.*, 2011 ▸; Meibohm *et al.*, 2014 ▸; Sellberg *et al.*, 2014 ▸). We have also developed a transmission-type liquid flow cell for XAS of liquid samples (Nagasaka *et al.*, 2020 ▸ *a*). The liquid layer is sandwiched between two Si_3_N_4_ membranes and the liquid thickness is controllable from 2000 nm to 20 nm by controlling the helium pressure around the liquid cell from 0.1 MPa to 0.12 MPa (Nagasaka *et al.*, 2018 ▸ *a*). In aqueous solutions, molecular interactions of solute organic molecules are measured by C and N *K*-edge XAS, and those of water are separately observed by O *K*-edge XAS (Nagasaka *et al.*, 2018*b* ▸, 2020 ▸ *b*). XAS is also applied to *in situ*/*operando* observations of catalytic (Yuzawa *et al.*, 2015 ▸) and electrochemical reactions (Nagasaka *et al.*, 2013 ▸, 2014 ▸).

On the other hand, the low-energy region below 200 eV includes important absorption edges for the investigation of chemical and biological phenomena such as the *K*-edges of Li and B and the *L*-edges of Si, P, S and Cl. In soft X-ray beamlines at synchrotron radiation facilities, monochromatic soft X-rays include not only first-order X-rays but also high-order X-rays due to the high-order diffraction of plane-grating monochromators. XAS measurement below 200 eV is difficult since transmitted soft X-rays mostly consist of high-order X-rays due to the low transmission of first-order X-rays (Chantler, 2000 ▸). For example, soft X-ray transmissions with photon energies of 160 eV, 190 eV, 400 eV and 530 eV are 11%, 22%, 82% and 93%, respectively, for a 1 cm propagation under atmospheric helium conditions. High-order X-rays are usually removed by metal filters, by energy analysis using a silicon drift detector (Lechner *et al.*, 1996 ▸), and by controlling the incident angle of a focused mirror in the beamline (Kitajima *et al.*, 1999 ▸). However, it is technically difficult to detect first-order X-rays below 200 eV without the contribution of high-order X-rays.

In this study, we propose the argon gas window for XAS in the low-energy region. Previously, the argon gas window would have been mainly used for the transmission of vacuum ultraviolet light (Mercier *et al.*, 2000 ▸; Giuliani *et al.*, 2011 ▸). It is also used as a band-pass filter that removes soft X-rays below 40 eV by filling with a small amount of argon gas (50 Pa) under vacuum conditions (Makimura *et al.*, 2006 ▸). On the other hand, the argon gas window is expected to obtain first-order X-rays in the low-energy region by removing the high-order X-rays above 240 eV with the absorption of the Ar *L*-edge (Sairanen *et al.*, 1996 ▸). Soft X-ray transmission measurements of helium and argon gas have been performed for the evaluation of the argon gas window.

## 2. Experimental methods   

**Figure 1:**
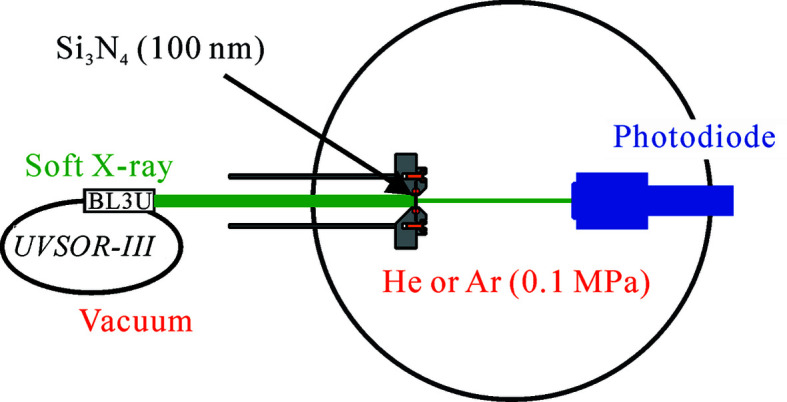
Schematic of the soft X-ray transmission measurements of helium or argon gas in the low-energy region. Details are given in the text.

Experiments were performed at the soft X-ray undulator beamline BL3U at the UVSOR-III synchrotron (Hatsui, 2004 ▸). Fig. 1 ▸ shows a schematic of the experimental setup. This system consists of two chambers under ultrahigh vacuum and atmospheric helium or argon conditions. The two chambers are separated by an Si_3_N_4_ membrane with a thickness of 100 nm and a window size of 200 µm × 200 µm (NTT-AT). The vacuum chamber is connected to the soft X-ray beamline. In the atmospheric chamber, helium or argon gas is flowed using a mass flow controller (Kofloc). Two gas samples can be mixed at any mixing ratio by the combination of two mass flow controllers. Soft X-rays under vacuum pass through the Si_3_N_4_ membrane window, the atmospheric helium or argon environments, and finally reach a photodiode detector (Opto Diode AXUV100G). The distance between the Si_3_N_4_ membrane and the photodiode detector is estimated to be 41 mm, which is sufficient to include our transmission-type liquid flow cell (Nagasaka *et al.*, 2018 ▸ *a*).

## 3. Results and discussion   

**Figure 2:**
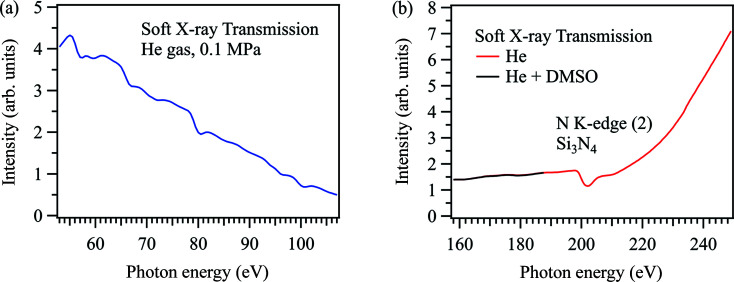
Soft X-ray transmission spectra of helium gas under atmospheric conditions in the energy region (*a*) from 53 eV to 107 eV and (*b*) from 158 eV to 249 eV. The transmission spectrum of helium gas mixed with DMSO gas is also shown.

Fig. 2 ▸ shows soft X-ray transmission spectra of helium gas in the low-energy region. Fig. 2 ▸(*a*) shows the energy region from 53 eV to 107 eV. The energy resolution is set to *E*/Δ*E* = 1000 at 100 eV. We expect to observe the double-excitation resonance state of helium gas (61 eV) (Domke *et al.*, 1996 ▸). We also expect to observe the Si *L*-edge XAS peak of the Si_3_N_4_ membrane (100 eV) (Tanaka *et al.*, 2001 ▸), which separates the atmospheric chamber from the beamline under ultrahigh vacuum conditions. However, the XAS peaks of both helium gas and the Si_3_N_4_ membrane are not observed in the transmission spectrum of helium gas. Although small peaks at 80 eV and 100 eV are observed, these peaks can be derived from the absorption of the Si_3_N_4_ membrane from the fifth- and fourth-order X-rays at the N *K*-edge (400 eV), respectively. The transmission spectrum of first-order X-rays is not obtained under atmospheric helium conditions since the ratio of the high-order X-rays is extremely high in this energy region.

Fig. 2 ▸(*b*) shows the soft X-ray transmission spectrum of helium gas in the energy region from 158 eV to 249 eV. The energy resolution is set to *E*/Δ*E* = 1000 at 200 eV. The absorption peak around 200 eV can be attributed to the absorption of second-order X-rays by the Si_3_N_4_ membrane at the N *K*-edge. On the other hand, we also measured soft X-ray transmission spectra of the mixture of di­methyl sulfoxide (DMSO) and helium gas, in which a small amount of DMSO gas is formed by bubbling liquid DMSO with helium gas. However, there are no spectral differences between DMSO/He mixed gas and pure helium gas in the S *L*-edge (170 eV), although the DMSO molecule contains a sulfur atom. The absorption of first-order X-rays is also not observed at 170 eV as the transmitted soft X-rays mostly consist of high-order X-rays. As a result, it is impossible to measure XAS under atmospheric helium conditions in the low-energy region below 200 eV.

**Figure 3:**
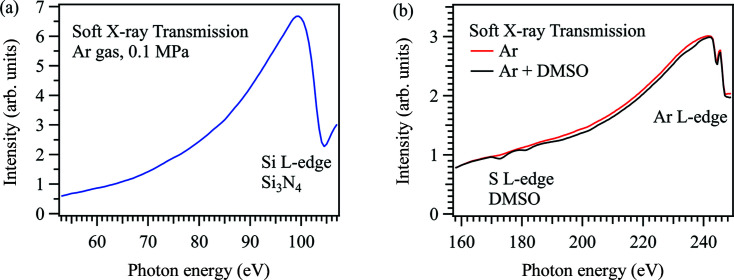
Soft X-ray transmission spectra of argon gas under atmospheric conditions in the energy region (*a*) from 53 eV to 107 eV and (*b*) from 158 eV to 249 eV. The transmission spectrum of argon gas mixed with DMSO gas is also shown.

Fig. 3 ▸ shows the soft X-ray transmission spectra of argon gas in the low-energy region. Fig. 3 ▸(*a*) shows the energy region from 53 eV to 107 eV. Argon gas has the role to remove high-order X-rays above 240 eV due to the absorption of the Ar *L*-edge. That is why the small peaks at 80 eV and 100 eV observed in Fig. 2 ▸(*a*) are completely diminished in the transmission spectrum of argon gas since these peaks are derived from the absorption of the Si_3_N_4_ membrane from the fifth- and fourth-order X-rays at the N *K*-edge. The spectrum also shows the absorption peak of the Si_3_N_4_ membrane around 104 eV at the Si *L*-edge. Fig. 3 ▸(*b*) shows the energy region from 158 eV to 249 eV. The absorption of argon gas is observed at 244 eV at the Ar *L*-edge. When a small amount of DMSO gas was mixed with the argon gas, we successfully observed the absorption peaks of DMSO gas at the S *L*-edge (170 eV), which clearly demonstrates the spectral difference between DMSO/Ar mixed gas and pure argon gas. Although the photon flux of the transmitted soft X-rays in argon gas is much smaller than that in helium gas (Chantler, 2000 ▸), the intensity change of the first-order X-rays is clearly observed. This is because soft X-rays above 240 eV are completely removed by argon gas due to the Ar *L*-edge.

**Figure 4:**
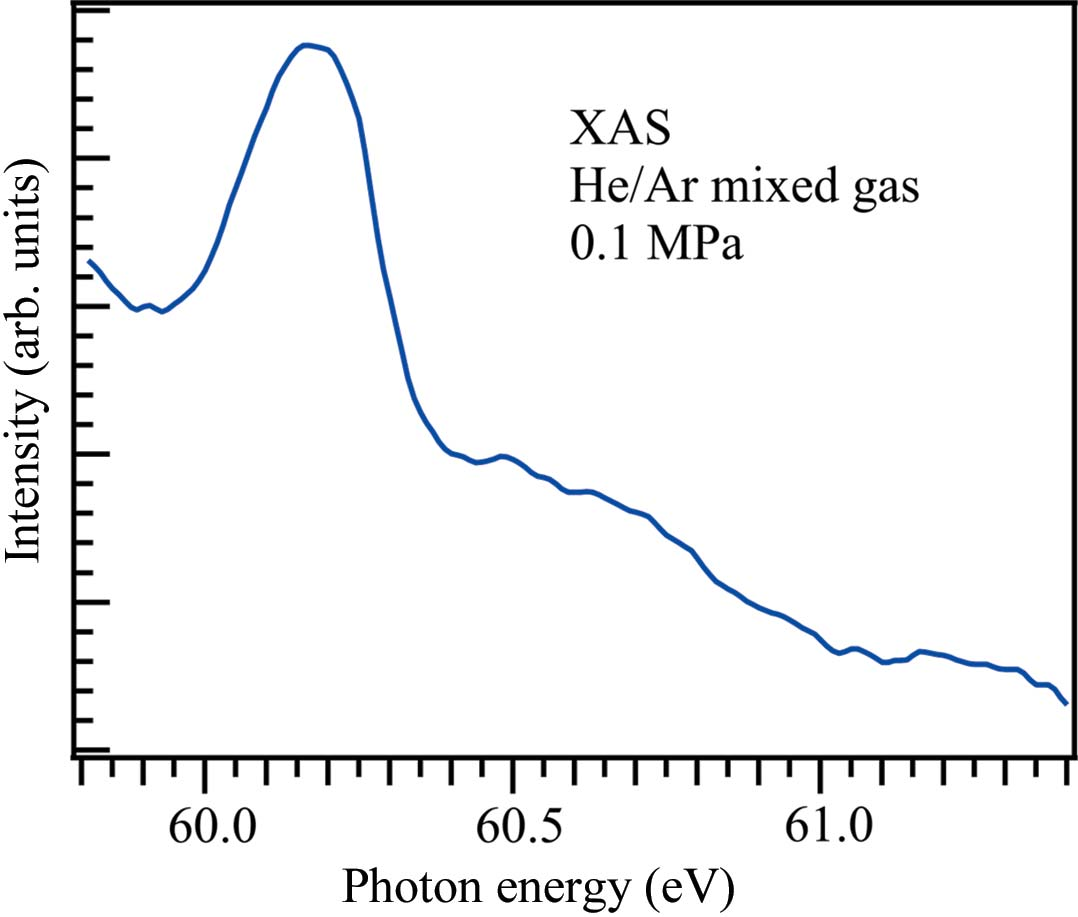
XAS spectrum of helium gas mixed with argon gas under atmospheric conditions.

Fig. 4 ▸ shows the XAS spectrum of helium gas, where a small amount of helium gas is mixed under atmospheric argon conditions. The energy resolution is set to *E*/Δ*E* = 1000 at 60 eV. The XAS spectrum is derived using the Beer–Lambert law, ln(*I*_0_/*I*), where *I*_0_ and *I* are the transmission signals of argon gas and Ar/He mixed gas, respectively. The spectrum shows the helium resonance energy *E*_2_ of the *n* = 2 double-excitation Rydberg series at 60.147 eV (Domke *et al.*, 1996 ▸). Since Li *K*-edge XAS spectra show peaks around 60 eV (O’Shaughnessy *et al.*, 2018 ▸), the measurement of Li *K*-edge XAS under atmospheric conditions is possible using the argon gas window. As a result, the argon gas window enables us to measure XAS under atmospheric conditions in the low-energy region from 60 eV to 240 eV.

## 4. Conclusions   

In this study, we confirm the effectiveness of the argon gas window as a new soft X-ray transmission window in the low-energy region below 200 eV, which includes chemically and biologically important absorption edges such as the *K*-edges of Li and B and the *L*-edges of Si, P, S and Cl. Since argon gas removes the high-order X-rays above 240 eV due to the absorption of the Ar *L*-edges, XAS under atmospheric conditions is possible in the low-energy region from 60 eV to 240 eV. We have successfully measured the double-excitation Rydberg series of helium gas (60 eV), Si *L*-edge XAS of the Si_3_N_4_ membrane (100 eV) and S *L*-edge XAS of DMSO gas (170 eV). We expect that the argon gas window will become the standard soft X-ray transmission window, as the helium gas window transmits soft X-rays above the C *K*-edge (280 eV) and the water window in the energy region between the C *K*-edge and the O *K*-edge (530 eV).

In our transmitted-type liquid flow cell, the liquid thickness is controlled by adjusting the helium pressure from 0.1 MPa to 0.12 MPa for XAS of liquid samples in transmission mode (Nagasaka *et al.*, 2018 ▸ *a*). When the buffer gas is changed to argon gas, we expect to be able to measure XAS of liquid samples in the low-energy region below 200 eV. XAS can be applied to the investigation of several catalytic and electrochemical reactions such as Li *K*-edge XAS of the lithium ion battery (Thackeray, 1997 ▸), S *L*-edge XAS of Nafion polymer films in a fuel cell electrode (Masuda *et al.*, 2013 ▸) and B *K*-edge XAS of the nickel borate electrocatalyst for oxygen evolution reactions (Dincă *et al.*, 2010 ▸). The application of biological phenomena is also expected, such as molecular interactions of phosphate ions with water in liquid bilayers around biological membranes (Tero *et al.*, 2017 ▸) from P *L*-edge XAS. Consequently, XAS in the low-energy region has the potential to enhance our understanding of physical, chemical and biological phenomena in solution, which would be realized by the argon gas window.
